# Three Amino Acid Changes in Avian Coronavirus Spike Protein Allow Binding to Kidney Tissue

**DOI:** 10.1128/JVI.01363-19

**Published:** 2020-01-06

**Authors:** Kim M. Bouwman, Lisa M. Parsons, Alinda J. Berends, Robert P. de Vries, John F. Cipollo, Monique H. Verheije

**Affiliations:** a Division of Pathology, Department of Pathobiology, Faculty of Veterinary Medicine, Utrecht University, Utrecht, The Netherlands; b Center for Biologics Evaluation and Research, Food and Drug Administration, Silver Spring, Maryland, USA; c Department of Chemical Biology and Drug Discovery, Utrecht Institute for Pharmaceutical Sciences, Utrecht University, Utrecht, The Netherlands; Loyola University Chicago

**Keywords:** coronavirus, infectious bronchitis virus, receptor-binding domain, receptors, spike protein, virus-host interactions

## Abstract

Infectious bronchitis virus (IBV) infects ciliated epithelial cells in the chicken respiratory tract. While some IBV strains replicate locally, others can disseminate to various organs, including the kidney. Here, we elucidate the determinants for kidney tropism by studying interactions between the receptor-binding domain (RBD) of the viral attachment protein spike from two IBV strains with different tropisms. Recombinantly produced RBDs from the nephropathogenic IBV strain QX and from the nonnephropathogenic strain M41 bound to the epithelial cells of the trachea. In contrast, only QX-RBD binds more extensively to cells of the digestive tract, urogenital tract, and kidneys. While removal of sialic acids from tissues prevented binding of all proteins to all tissues, binding of QX-RBD to trachea and kidney could not be blocked by preincubation with synthetic alpha-2,3-linked sialic acids. The lack of binding of QX-RBD to a previously identified IBV-M41 receptor was confirmed by enzyme-linked immunosorbent assay (ELISA), demonstrating that tissue binding of QX-RBD is dependent on a different sialylated glycan receptor. Using chimeric RBD proteins, we discovered that the region encompassing amino acids 99 to 159 of QX-RBD was required to establish kidney binding. In particular, QX-RBD amino acids 110 to 112 (KIP) were sufficient to render IBV-M41 with the ability to bind to kidney, while the reciprocal mutations in IBV-QX abolished kidney binding completely. Structural analysis of both RBDs suggests that the receptor-binding site for QX is located at a different location on the spike than that of M41.

**IMPORTANCE** Infectious bronchitis virus is the causative agent of infectious bronchitis in chickens. Upon infection of chicken flocks, the poultry industry faces substantial economic losses by diminished egg quality and increased morbidity and mortality of infected animals. While all IBV strains infect the chicken respiratory tract via the ciliated epithelial layer of the trachea, some strains can also replicate in the kidneys, dividing IBV into the following two pathotypes: nonnephropathogenic (example, IBV-M41) and nephropathogenic viruses (including IBV-QX). Here, we set out to identify the determinants for the extended nephropathogenic tropism of IBV-QX. Our data reveal that each pathotype makes use of a different sialylated glycan ligand, with binding sites on opposite sides of the attachment protein. This knowledge should facilitate the design of antivirals to prevent coronavirus infections in the field.

## Abstract (precis)

Infectious bronchitis virus is the causative agent of infectious bronchitis in chickens. Upon infection of chicken flocks, the poultry industry faces substantial economic losses by diminished egg quality and increased morbidity and mortality of infected animals. While all IBV strains infect the chicken respiratory tract via the ciliated epithelial layer of the trachea, some strains can also replicate in the kidneys, dividing IBV into the following two pathotypes: nonnephropathogenic (example, IBV-M41) and nephropathogenic viruses (including IBV-QX). Here, we set out to identify the determinants for the extended nephropathogenic tropism of IBV-QX. Our data reveal that each pathotype makes use of a different sialylated glycan ligand, with binding sites on opposite sides of the attachment protein. This knowledge should facilitate the design of antivirals to prevent coronavirus infections in the field.

## INTRODUCTION

Infectious bronchitis is a disease in chickens caused by infectious bronchitis virus (IBV). In the poultry industry, infection of chicken flocks with IBV causes economic losses by reducing egg quantity and quality. In addition, animals become more susceptible to secondary bacterial infections like Escherichia coli (1). The severity of disease and organs affected depend primarily on the IBV strain (2). Phylogenetic classification of IBV strains results in 32 phylogenetic lineages (GI-1 to GI-27 and GII to GVI) (3), of which GI-1 includes historically the first IBV genotype identified, Massachusetts (IBV-Mass). IBV-Mass infections are reported worldwide, and in Europe, GI-1 is currently the 3rd most prevalent genotype (2). The more prevalent IBV genotype circulating in Europe is IBV-QX (GI-19) (2, 3), which has been reported to cause kidney disease in contrast to IBV-Mass (2).

IBV primarily infects the respiratory tract, where the virus can bind and infect the ciliated epithelial lining of the trachea (4, 5). Upon infection of IBV, clinical symptoms such as snicking, wheezing, and/or nasal discharge are reported (6). While infection of IBV-Mass (of which strain M41 is the prototype) is predominantly detected in the upper respiratory tract (7) including the trachea (2), replication of IBV-QX is additionally found in the kidneys (7–9), oviduct, and the gastrointestinal tract (10, 11), leading to additional clinical symptoms like swollen proventriculus (12) and reduction of egg production (13, 14). Because of these additional clinical symptoms, IBV-QX is described as a nephropathogenic IBV strain (2).

Binding to host tissues is the first step in the viral life cycle of IBV and therefore a critical factor in determining tissue tropism. Tissue tropism differs based on the amino acid composition of the spike protein as shown by recombinantly produced proteins (15–17) and infection assays with recombinant viruses (18). The spike of IBV is posttranslationally cleaved into two subunits, S1 and S2, where S2 is anchored in the virus membrane and important for membrane fusion. S1 comprises the head domain of spike and is responsible for host receptor binding (19). Using recombinantly expressed M41-S1 proteins, alpha-2,3-linked sialic acids were identified as the IBV receptor on a glycan array, where specific binding to the ligand Neu5Acα2-3Galβ1-3GlcNAc was observed (19). Recently the cryo-electron microscopy (cryo-EM) structure of the M41 spike has been resolved (20), indicating that the S1 subunit consists of two independent folding domains, the N-terminal domain (NTD) (amino acids 21 to 237) and C-terminal domain (CTD) (amino acids 269 to 414), with a proposed receptor-binding site in both domains. Experimental evidence using recombinantly expressed spike domains has indicated that amino acids 19 to 272 of the M41 spike are sufficient for binding to trachea as well as binding to alpha-2,3-linked sialic acids (15). This domain thus contains a receptor-binding domain (RBD) and can be used to study the biological implications of genetic variation in circulating IBV genotypes.

In this study, we set out to identify how genetic variations in IBV spike proteins have contributed to different host tropisms. We demonstrate that QX-RBD binding to trachea and kidney is dependent on a different sialylated glycan ligand compared to M41-RBD. In particular, introduction of amino acids 110 to 112 (KIP) of the QX spike into M41-RBD was sufficient to extend its tropism toward the kidney. Previous docking experiments (17) and structural analysis suggest that the binding pockets for the different glycans are located at opposite sites of each spike protein.

## RESULTS

### The N-terminal domain of IBV-QX spike contains a receptor-binding domain.

Eighty-five percent of the amino acids between the sequences of the first 257 amino acids of IBV-QX and IBV-M41 are either identical or similar. Here, we set out to determine which of the dissimilar amino acids are the determinants for the difference in tissue tropism.

**FIG 1:**
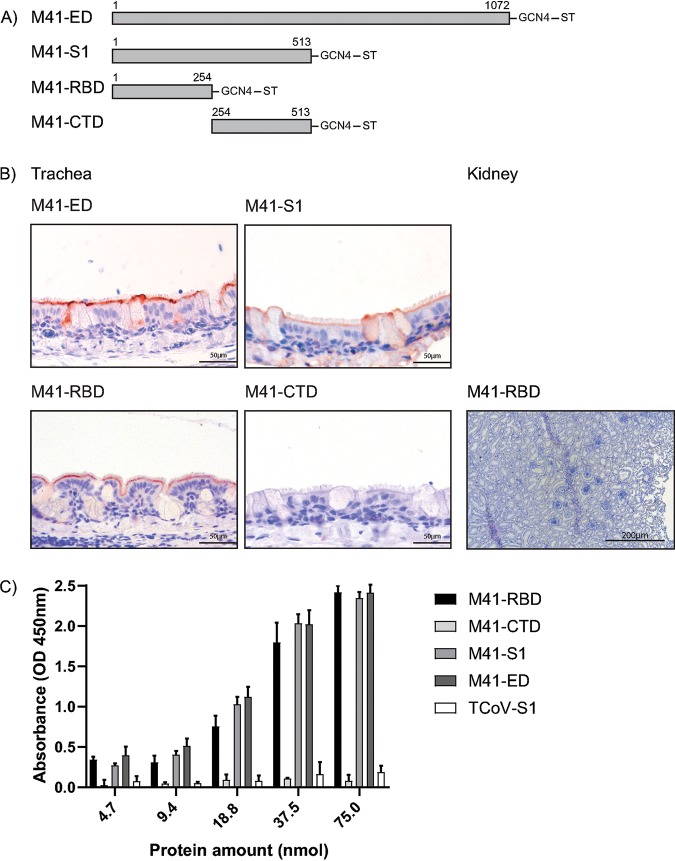
Binding of M41 spike proteins to paraffin-embedded healthy chicken trachea and kidney tissue. (A) Schematic representation of spike proteins, M41-ectodomain (ED) (amino acids 1 to 1072), M41-S1 (amino acids 1 to 513), M41-RBD (amino acids 1 to 254), and M41-CTD (amino acids 254 to 513) followed by a trimerization domain (GCN4) and strep-tag (ST). Numbering starts at 1 of the mature protein sequence. (B) Protein binding of M41 spike proteins observed in the trachea and kidney is visualized by red staining. (C) Affinity of M41 spike proteins for the known ligand (Neu5Acα2-3Galβ1-3GlcNAc) in solid-phase ELISA. At all protein amounts, a significant difference of at least *P* < 0.01 was observed between M41-RBD, M41-S1, M41-ED, and M41-CTD and TCoV-S1, which served as a negative control tested in two-way ANOVA. ELISA was performed in triplicate where average and standard deviations are shown.

In previous work, we demonstrated the M41-RBD was sufficient to bind to chicken trachea (15). To verify that no additional sites are present in M41 that could bind to kidney or trachea tissue, we produced recombinant proteins consisting of the full ectodomain (ED), the S1 portion of the ED, the RBD (NTD of S1), and the CTD of S1. Each protein was assessed for binding using trachea and kidney tissue slides. Binding to trachea tissue was observed using M41-ED, S1, and RBD but not CTD to ciliated epithelium of the trachea, specifically located at the base of the cilia (Fig. 1), confirming previous observations (15, 19, 21). None of the proteins bound kidney tissue, which is shown by a representative picture using M41-RBD (Fig. 1B). Binding affinity to the known ligand (Neu5Acα2-3Galβ1-3GlcNAc) in enzyme-linked immunosorbent assay (ELISA) was observed using M41-RBD, M41-S1, and M41-ED, not significantly different when compared to each other but significantly higher compared to those of M41-CTD and turkey coronavirus (TCoV)-S1 (Fig. 1C). These results indicate that ligand binding of M41-RBD is not significantly different compared to that of M41-S1 and M41-ED, suggesting no additional ligand-binding motifs are present in S1 and ED; thus, in the remaining experiments, we used M41-RBD, as the tissue tropism of the virus is reflected using this recombinant protein.

**FIG 2:**
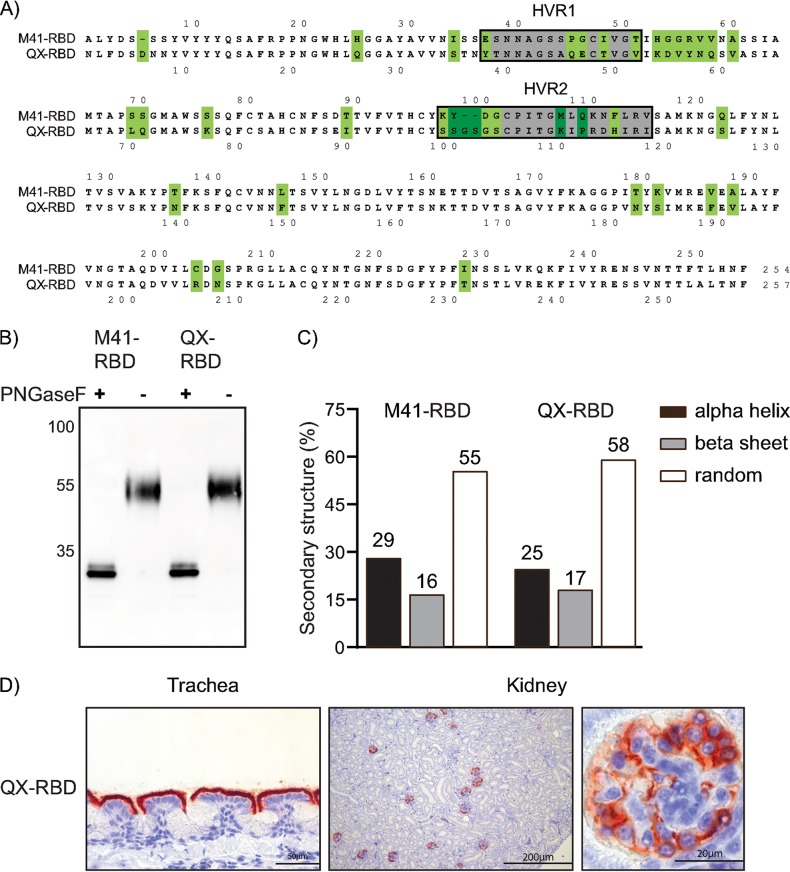
IBV M41- and QX-RBD protein analysis. (A) Amino acid alignment of M41-RBD (amino acids 19 to 272; GenBank accession number AY851295) and amino acids 19 to 275 (GenBank accession number AFJ11176) of the QX spike. Numbering starts at 1 of the mature protein sequence (signal sequence not shown). Dots indicate identical amino acids. Gray highlights surrounded by a black box indicate previously identified hypervariable regions of IBV-Mass (22). Green highlights indicate very different residues. (B) M41- and QX-RBD with and without pretreatment of PNGase F analyzed by Western blotting using Strep-Tactin HRP antibody. (C) Percentage of secondary protein structures calculated based on CD analysis of M41- and QX-RBD. (D) Binding of QX-RBD to paraffin-embedded healthy chicken trachea and kidney visualized by red staining in protein histochemistry.

Amino acid alignment of the mature protein sequence of the receptor-binding domain (RBD) of M41 and a comparable size fragment of the QX spike displayed a sequence identity of 73.6% (Fig. 2A), with the highest sequence diversity between amino acids 37 to 60 and 98 to 115. These regions include the previously described hypervariable regions (HVRs) (highlighted in gray) of M41-S1 (22). Before studying whether sequence diversity between the RBDs of M41 and QX contributes to the reported broader tropism of QX *in vivo*, we first determined if the potential RBD of QX behaved like that of M41 (Fig. 1) and that it contains a receptor-binding domain (15). Both proteins were produced as soluble recombinant protein in mammalian cells and analyzed on Western blots after purification. Before loading, a fraction was pretreated with peptide-*N*-glycosidase F (PNGase F) to remove posttranslational glycosylation. QX- and M41-RBD migrated comparably at around 55 kDa (including glycosylation) and had a backbone of around 32 kDa as expected after PNGase F treatment (Fig. 2B). Circular dichroism (CD) spectroscopy was used to assess similarities in secondary structure between M41- and QX-RBD. Spectra at all temperatures followed the same curve, and both proteins had similar broad melting curves, indicating that both proteins are equally stable (data not shown). Subsequent secondary structure calculations using DichroWeb (23) presented that M41- and QX-RBD contain 29 and 25% α-helix, 16 and 17% β-strands, and 55 and 58% random structures, respectively (Fig. 2C). Finally, we confirmed that the QX-RBD was biologically active by applying it to chicken trachea tissue slides in protein histochemistry. We observed clear binding to the ciliated lining of epithelial cells and structures present in the kidney (Fig. 2D), indicating that QX-RBD, like M41-RBD, contains a receptor-binding site.

### QX-RBD shows a broader tissue tropism than that of M41-RBD.

Next, we used M41- and QX-RBDs to study the distribution of host attachment factors across chicken tissues. To this end, we allowed both proteins to bind to tissue microarray slides containing 28 different chicken tissues (24). Binding of M41-RBD was primarily found on the ciliated lining of the epithelium of the proximal and distal trachea (Fig. 1), but additional staining was observed in the epithelial lining of the colon, cecal tonsil, ureter, oviduct, and conjunctiva (Table 1). QX-RBD bound to the same tissues as M41-RBD, but additional binding was observed in gizzard, ileum, and cloaca of the digestive tract, as well as liver and kidneys (Table 1 and Fig. 2D), reflecting that observed *in vivo* for replication of both genotypes. Detailed analysis of staining present in the kidney showed that binding of QX-RBD was restricted to the parietal epithelium of Bowman’s capsule in the glomerulus (Fig. 2D). No binding to the glomeruli was observed when using M41-RBD in three independent experiments using different protein batches. Taken together, QX-RBD shows a markedly broader binding profile than that of M41-RBD, which is in line with the reported broader tissue tropism *in vivo* (2).

**TABLE 1:** Relative binding of IBV RBD proteins to paraffin-embedded healthy chicken tissues

Tissue	QX-RBD^*a*^	M41-RBD^*a*^
Nostril		
Proximal trachea	+	+
Distal trachea	+	+
Lung		
Esophagus		
Gizzard	+	
Proventriculus		
Duodenum		
Ileum	+	
Colon	+	+
Cecal tonsil	+	+
Spleen		
Liver	+−	
Adrenal glands		
Pancreas		
Kidney	+	
Ureter	+	+
Heart		
Skin		
Conjunctiva	+	+
Muscles		
Ovary		
Oviduct	+	+
Cloaca	+−	
Cerebrum		
Cerebellum		
Brain stem		
Sciatic nerve		

a White, no visible staining; +**−**, staining of a few cells; +, staining of most epithelial cells.

### QX-RBD binds to sialic acids on chicken tissues.

**FIG 3:**
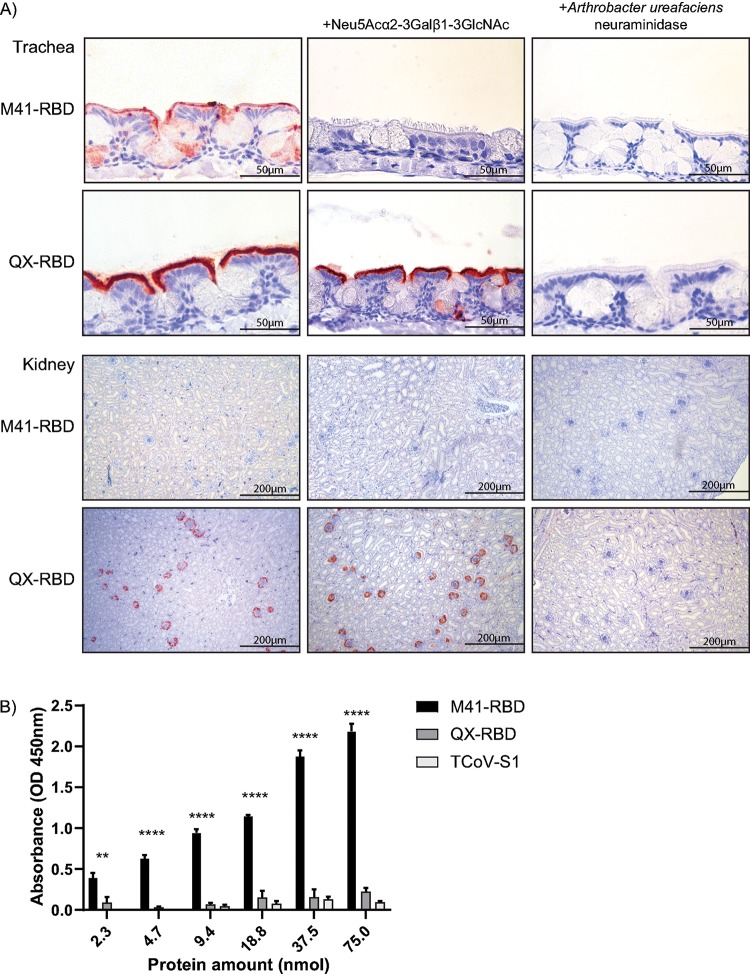
Avidity and affinity of M41- and QX-RBD for host factors. (A) Protein histochemistry of M41- and QX-RBD onto paraffin-embedded chicken trachea and kidney tissue (left column) upon preincubation of proteins with Neu5Acα2-3Galβ1-3GlcNAc (middle column) or pretreatment of tissues with Arthrobacter ureafaciens neuraminidase (right column). (B) Affinity of RBD proteins for Neu5Acα2-3Galβ1-3GlcNAc in ELISA. **, *P* < 0.01; ****, *P* < 0.001 tested in two-way ANOVA. TCoV-S1 was used as a negative control in equal molar amounts. ELISA was performed in triplicate with all proteins; average is shown with standard deviations.

To investigate whether the expanded tropism of QX-RBD can be explained by binding with similar specificity, but higher affinity, to the previously identified M41 receptor (19), we preincubated both RBD proteins with the synthetic Neu5Acα2-3Galβ1-3GlcNAc before applying them to trachea and kidney tissue slides. As expected, binding of M41-RBD to the trachea was completely prevented (Fig. 3A, middle column) in the presence of the synthetic M41 ligand. In contrast, QX-RBD still showed strong binding to the ciliated epithelium of the trachea and glomeruli of the kidney. To confirm the loss of binding of QX-RBD to Neu5Acα2-3Galβ1-3GlcNAc, a solid-phase ELISA was performed, in which Neu5Acα2-3Galβ1-3GlcNAc was coated. As expected, no binding of QX-RBD to this particular glycan was observed at any of the protein concentrations, which is comparable to that of the negative control TCoV-S1 (only binding longer branched galactose-terminated glycans [25]), while M41-RBD bound to Neu5Acα2-3Galβ1-3GlcNAc in a concentration-dependent manner (Fig. 3B).

To reveal whether QX-RBD exclusively depends on sialic acids, trachea and kidney tissue slides were pretreated with Arthrobacter ureafaciens neuraminidase before applying M41- and QX-RBD. Removal of sialic acids from trachea and kidney tissue completely prevented binding of both RBD proteins (Fig. 3A, right column), indicating that QX-RBD binding is dependent on the presence of sialic acids on host tissues.

### M41-RBD gains kidney binding upon MLQ107-109KIP mutation.

**FIG 4:**
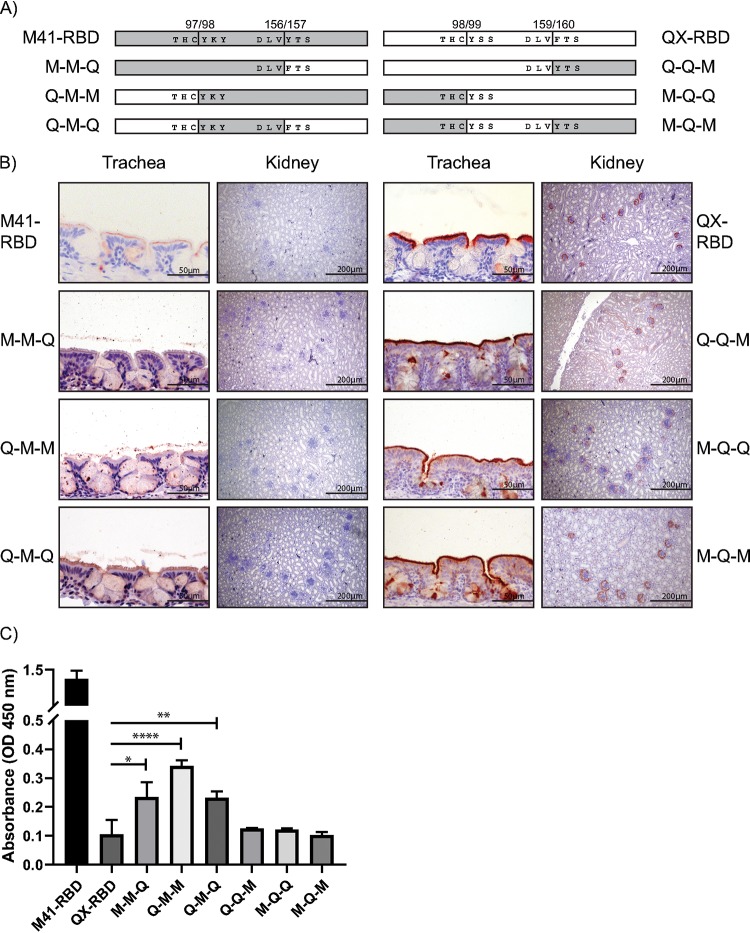
Chimeric RBD protein binding to chicken tissues. (A) Schematic representation of chimeric RBD proteins. The gray box indicates M41, and the white box indicates the QX wild-type sequence. Amino acids surrounding the transitions between the different domains of the chimeric proteins are indicated including the amino acid number of the wild-type sequence. (B) Binding of chimeric RBDs to trachea and kidney tissue in protein histochemistry. (C) Affinity of 37.5 nmol chimeric RBD proteins in ELISA for Neu5Acα2-3Galβ1-3GlcNAc. *, *P* < 0.05; **, *P* < 0.01; ****, *P* < 0.001, tested in two-way ANOVA. ELISA was performed with all chimeric RBD proteins in triplicates.

To gain in-depth knowledge on the interaction of the IBV RBD proteins and chicken tissue, we set out to determine the critical amino acids of viral spike proteins involved in binding to these glycan receptors, thereby leading to the ability to bind to kidney tissue. Chimeric RBD proteins were generated by dividing each wild-type RBD into three domains and mixing them to get six different combinations (schematic representations in Fig. 4A). These chimeras were then applied to trachea and kidney tissue slides. Chimeric proteins containing amino acids 98 to 156 (middle domain) of M41 (M-M-Q, Q-M-M, and Q-M-Q) demonstrated reduced binding to trachea and no detectable binding to kidney tissue (Fig. 4B). In contrast, chimeric proteins containing this region of QX (Q-Q-M, M-Q-Q, and M-Q-M) had comparable binding to tissues as QX-RBD. In particular, strong binding to the ciliated epithelial lining of trachea and specific staining in Bowman’s capsule in the glomerulus was observed (Fig. 4B). Like wild-type RBDs, binding of all chimeric proteins was dependent on the presence of sialic acids, as pretreatment of host tissues with AUNA abrogated binding (data not shown). M-M-Q, Q-M-M, and Q-M-Q proteins had reduced affinity for Neu5Acα2-3Galβ1-3GlcNAc (Fig. 4C), potentially explaining the reduced staining of these proteins to trachea tissue (Fig. 4B). None of the RBD proteins containing the middle QX sequence (Q-Q-M, M-Q-Q, and M-Q-M) had affinity for this glycan in the ELISA as expected based on tissue staining (Fig. 4B and C), which is in line with the hypothesis these proteins are dependent on binding to the QX receptor instead of the known M41 receptor. These results indicate that the receptor-binding site responsible for recognition of the QX receptor is determined by amino acids 99 to 159 of the spike.

**FIG 5:**
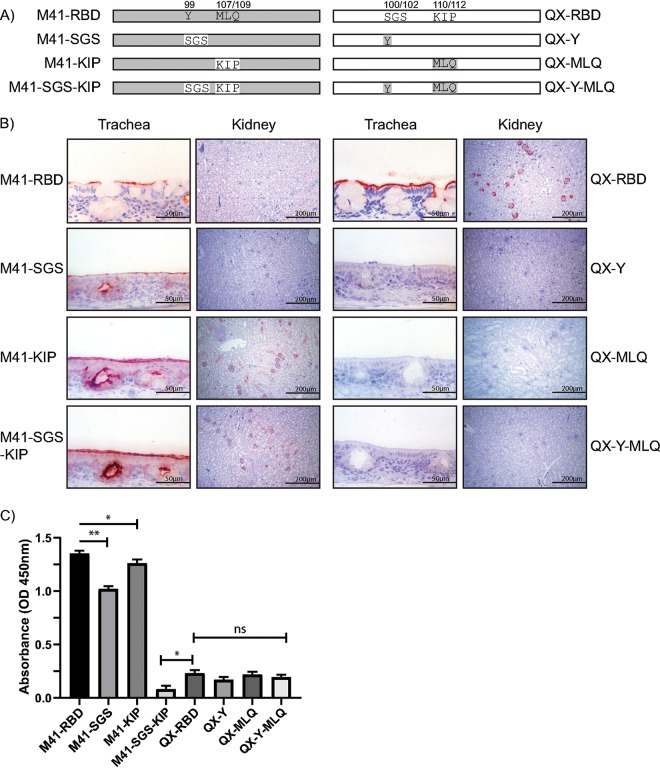
Identification of amino acids involved in IBV kidney binding using chimeric proteins. (A) Schematic representation of chimeric RBD proteins. The gray box indicates M41-, and the white box indicates the QX wild-type sequence. Numbers above indicate the positions of the amino acid triplicates swapped between M41 and QX. (B) Binding of chimeric RBDs to trachea and kidney tissue in protein histochemistry. (C) Binding of chimeric RBD proteins (37.5 nmol) in ELISA to Neu5Acα2-3Galβ1-3GlcNAc. *, *P* < 0.05; **, *P* < 0.01, tested in two-way ANOVA. ELISA was performed with all chimeric RBD proteins in triplicates.

To ultimately determine the critical residues of the RBD for the interaction with chicken kidney tissue, additional chimeric proteins were produced and used in protein histochemistry. We exchanged two triplets (highlighted in dark green in Fig. 2A) of amino acids in HVR 2 (amino acids 99 to 115 of M41), either alone or in combination, that had the high diversity in amino acid characteristics (schematic representations in Fig. 5A). Introduction of the M41 sequence in the QX-RBD protein, SGS100–102Y (QX-Y) and KIP110–112MLQ (QX-MLQ) and their combination (QX-Y-MLQ), all resulted in a loss of binding to trachea and kidney tissues (Fig. 5B, right). In contrast, introduction of MLQ107–109KIP into M41-RBD (M41-KIP) resulted in binding to glomeruli in kidney tissue, both in a wild-type background and in the Y99SGS (M41-KIP-SGS) mutant (Fig. 5B, left). In the ELISA, both M41-SGS and M41-KIP demonstrated a decreased affinity for alpha-2,3-linked sialic acids compared to that of M41-RBD, while introduction of both triplets SGS and KIP (M41-SGS-KIP) completely abolished binding to this glycan (Fig. 5C). Taken together, these results suggest that a receptor-binding site critical to establish kidney binding requires amino acids KIP at position 107 to 109 in M41-RBD.

### Receptor-binding site of the QX-specific receptor differs from that proposed for M41.

**FIG 6:**
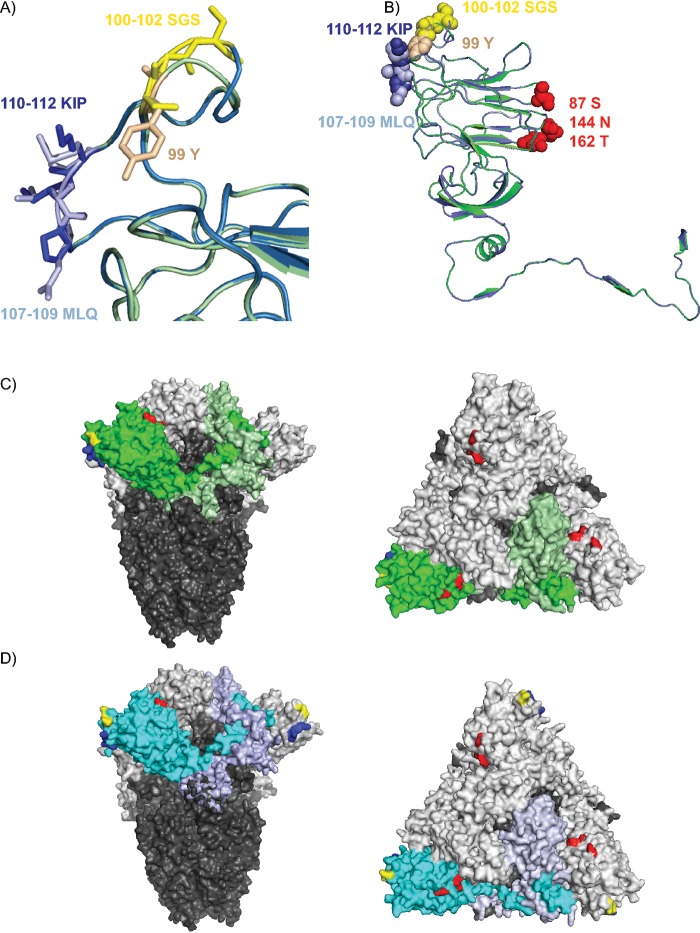
Model of IBV spike with predicted receptor-binding sites. (A) Structural alignment overlay of QX-RBD (blue ribbon) onto M41-RBD (green ribbon), based on PDB accession number 6cv0 (20) using Swiss-Model. Detailed representation of the receptor-binding site identified for QX-RBD; indicated in yellow sticks are amino acids 100 to 102 SGS in QX-RBD and in beige 99 Y in M41-RBD. 110 to 112 KIP of QX-RBD is indicated with dark blue sticks, whereas light blue represents 107 to 109 MLQ in M41-RBD. (B) Amino acids involved in receptor binding of IBV. Blue ribbon represents the modeled QX-RBD structure with amino acids 100 to 102 (SGS) as yellow spheres and 110 to 112 (KIP) as blue spheres. The green ribbon represents the M41-RBD with 99 (Y) as beige spheres and 107 to 109 (MLQ) as light blue spheres. Amino acids in red spheres (S87, N144, and T162) are previously predicted to be involved in alpha-2,3-linked sialic acid binding of M41-RBD (17). (C) Surface representation of the trimeric M41 spike cryo-EM structure (20). S2 is in dark gray for all monomers. S1 is in light gray with one S1 monomer colored bright green for the RBD domain and pale green for the CTD. Amino acids involved in ligand binding are highlighted as follows: yellow is 99Y (100 to 102 SGS in QX), dark blue is 107 to 109 MLQ (110 to 112 KIP in QX), and red is S87, N144, and T162. (D) Modeled QX spike based on PDB accession number 6cv0, colors as indicated in panel C, except the S1 of QX is blue, and the RBD is bright blue. Representations on right of panels C and D are structures turned 90 degrees toward the viewer. All representations were made using PyMOL viewer.

Finally, we modelled QX-RBD based on a structural overlay with the recently resolved cryo-EM structure of the M41 spike (20) and focused on the amino acids allowing kidney binding. The overall structure of both proteins is comparable (Fig. 6A, green ribbon, M41; blue ribbon, QX); however, the loop consisting of HVR 2 is slightly larger in QX-RBD as expected, as there are two additional amino acids present (Fig. 6A, SGS100–102 for QX-RBD versus Y99 in M41-RBD). Interestingly, this loop was predicted to be involved in sugar binding (20), which we showed to be true for QX-RBD but not for M41-RBD. In detail, the tyrosine (Y99) in the M41 structure (Fig. 6A, beige) occupies more space than serine (S in QX) and can be seen reaching toward a neighboring loop. Furthermore, the 110–112KIP sequence identified in QX-RBD (Fig. 6A, dark blue) places a positive charge at the protein surface, which is not present in 107–109MLQ in M41-RBD (Fig. 6A, light blue). Previous *in silico* docking analysis performed with potential alpha-2,3-linked ligands to the M41-RBD protein identified amino acids S87, N144, and T162 to potentially be involved in receptor binding (17). When we highlighted these amino acids predicted to be involved in binding to alpha-2,3-linked sialic acids (Fig. 6B, red spheres) and the amino acid triplicates involved in binding to the QX-specific receptor (100 to 102 [SGS, yellow spheres] and 110 to 112 [KIP, dark blue spheres]) in the overlaid RBD ribbon structure, we demonstrate that binding of the different ligands recognized by M41 and QX are on different sides of the protein (Fig. 6B). Furthermore, when these amino acids were highlighted in the full cryo-EM resolved structures of M41 (Fig. 6C) and QX (Fig. 6D), it clearly shows that the potential ligand-binding site of M41 is at a different location compared to the QX ligand-binding site (Fig. 6C and D).

In conclusion, we demonstrate that IBV-QX recognizes a sialylated glycan receptor present on chicken tissues that differs from that recognized by M41 and that this binding is likely required for the extended *in vivo* tissue tropism of the virus.

## DISCUSSION

In this study, we reveal that nephropathogenic IBV-QX shows expanded binding tropism based on interactions with sialic acid(s) on chicken tissues that differs from the receptor elucidated for IBV-M41. Using chimeric proteins and *in silico* modeling, we conclude that amino acids in hypervariable region 2 are critical for recognizing such a sialylated glycan receptor.

The N-terminal domain of IBV-QX spike protein comprises, like previously shown for M41 (15), a receptor-binding domain. Interestingly, QX-RBD shows no affinity for the known ligand of M41 (Neu5Acα2-3Galβ1-3GlcNAc) in glycan ELISA, while it gained binding to a novel unidentified sialylated glycan receptor. Other avian gammacoronaviruses, including guinea fowl and turkey coronavirus are dependent on long glycans (linear or branched) capped with either an alpha-2,6-linked sialic acid (GfCoV only) or galactose ending glycans (both TCoV and GfCoV) (25, 26). Viruses of other coronavirus genera are dependent on sialic acid receptors, like alpha coronaviruses transmissible gastroenteritis virus (TGEV) and porcine epidemic diarrhea virus (PEDV) (27, 28) and beta coronaviruses human coronavirus (HCoV)-OC43 and bovine coronavirus (BCoV) (29). Whether other nephropathogenic IBV strains are dependent on the same sialic acid receptors for binding and subsequent infection as QX remains to be determined.

To elucidate the specific ligand used by QX-RBD, we performed several binding studies using previously developed glycan arrays (30, 31) containing multiple linear and branched glycans capped without or with alpha-2,3-linked sialic acids or alpha-2,6-linked sialic acids. Unfortunately, no binding was observed using our RBD proteins. This may be explained by the usage of RBD proteins instead of the full S1, as used previously (19), or by the composition and fine structure of the glycans present in both arrays. On the arrays used, most glycans contain the linkage found in mammals (Galβ1,4GlcNAc), while the minority contain a Galβ1,3GlcNAc linkage. The exact nature of the receptor recognized by IBV-QX could be a more complex glycan containing a Galβ1,3GlcNAc that is scarcely populated on glycan arrays.

Comparison of the spikes of various IBV strains with reported nephropathogenicity, including IBV clade GI-14 (including strain B1648 [3]) and clade GI-13 (including strain 793B [3]), shows that only nephropathogenic IBV clades contain an amino acid triplicate at position 100 to 102, whereas in IBV-Mass genotypes, 99Y/H is expressed, thereby shortening HVR 2 with two amino acids. Sequence alignment of this amino acid triplicate (100 to 102 in QX-RBD) varies in nephropathogenic IBV genotypes from SGS/SGT for clade GI-19 (IBV-QX), NQQ/SQQ for clade GI-13 (IBV-793B), and SGA for clade GI-14 (IBV-B1648) at that position. Furthermore, the amino acid triplet 110 to 112 KIP is not conserved across IBV genotypes. In these genotypes, amino acid triplets LIQ for B1648 and MIP for 793B are present, which are sequence combinations of amino acids found in Mass (clade GI-1) and QX (clade GI-19). In terms of hydrophobicity and size, amino acid triplet MLQ (M41) is very similar to LIQ (B1648), whereas the proline (P) in KIP (QX) and MIP (793B) reduces the flexibility of the loop.

Structural analysis of the RBD of IBV suggests that the receptor-binding sites for M41 and QX are positioned at different sides of the RBD (Fig. 6B through D). Previous *in silico* predictions of the interaction with alpha-2,3-linked sialic acid ligands in M41-RBD pointed toward three amino acids, S87, N144, and T162, which are in close proximity to four essential *N*-glycosylation sites (N33, N59, N85, and N160) (17). Although the amino acid sequence of S87, N144, and T162 is conserved between M41 and QX, one of the essential *N*-glycosylation sites is at a different position (N59 in M41 and N58 in QX). This may result in a different conformation of the ligand-binding site, thereby preventing QX-RBD wild-type binding to the M41 ligand, which was supported by experimental evidence using the chimeric M41-RBD protein where this glycosylation site was replaced, resulting in loss of binding to trachea tissue (data not shown). Furthermore, in the publication where the cryo-EM structure of M41 was resolved, the loop consisting of amino acids present in HVR 2 of the spike was proposed to be required for receptor binding (20). Our data points toward involvement of the unglycosylated loop containing HVR 2 for recognition of the QX glycan ligand but not the M41 ligand. Furthermore, the cryo-EM structure of the M41-CTD predicts other putative receptor-binding motif loops in M41 spike (20). In Fig. 1, we demonstrated that no binding to trachea and kidney tissue was observed using our recombinantly expressed M41-CTD, in contrast to their published results. As binding of QX-RBD reflects the tissue tropism of QX-infected birds, we speculate whether these loops (in the CTD) are necessary for initial receptor recognition and are involved in QX infection.

In conclusion, we demonstrated that IBV-QX binding to chicken trachea and kidney tissue is dependent on a sialylated glycan receptor and that amino acids in HVR 2 of the QX-RBD are critical for this receptor-binding profile. This knowledge adds to our understanding of differences in tissue tropism between IBV strains *in vivo* and may contribute to designing new antivirals to prevent coronavirus infections in the field.

## MATERIALS AND METHODS

### Construction of the expression plasmids.

The expression plasmids containing the codon-optimized M41-ED (amino acids 19 to 1091 [21]), M41-S1 (amino acids 19 to 532 [19]), M41-RBD (amino acids 19 to 272 [15]), and M41-CTD (amino acids 273 to 532 [15]; GenBank accession number AY851295) sequences followed by a trimerization domain (GCN4) and strep-tag (ST) were described previously (15). The codon-optimized sequence of QX-RBD (amino acids 19 to 275; GenBank accession number AFJ11176), containing upstream NheI and downstream PacI restriction sites, was obtained from GenScript and cloned into the pCD5 expression vector by restriction digestion as previously described (19). Fragments to generate chimeric RBD proteins were created by splice overlap extension PCR using the primers in Table 2 and cloned into the pJET vector (Thermo Scientific, USA). The sequences were verified by automated nucleotide sequencing (Macrogen, The Netherlands) before cloning each fragment into the pCD5 expression vector. Mutations up to 9 nucleotides (nt) were introduced by site-directed mutagenesis using the primers listed in Table 2, and the sequences were subsequently verified by automated nucleotide sequencing (Macrogen, The Netherlands).

**TABLE 2:** Primer sequences to create chimeric RBD plasmids^*a*^

Protein	Original plasmid	Forward primer^*b*^	Reverse primer^*b*^
MMQ	M41-RBD	gtcgcttccgtgctagca	acaccagrtckccgttcag
	QX-RBD	ctgaacggmgayctggtgt	ctgcttcatgcgcttaattaa
		gtcgcttccgtgctagca	ctgcttcatgcgcttaattaa
MQM	MQQ	gtcgcttccgtgctagca	acaccagrtckccgttcag
	M41-RBD	ctgaacggmgayctggtgt	ctgcttcatgcgcttaattaa
		gtcgcttccgtgctagca	ctgcttcatgcgcttaattaa
MQQ	M41-RBD	gtcgcttccgtgctagca	tagcaatgwgtsacgaacactg
	QX-RBD	cagtgttcgtsacwcattgcta	ctgcttcatgcgcttaattaa
		gtcgcttccgtgctagca	ctgcttcatgcgcttaattaa
QQM	QX-RBD	gtcgcttccgtgctagca	acaccagrtckccgttcag
	M41-RBD	ctgaacggmgayctggtgt	ctgcttcatgcgcttaattaa
		gtcgcttccgtgctagca	ctgcttcatgcgcttaattaa
QMQ	QMM	gtcgcttccgtgctagca	acaccagrtckccgttcag
	QX-RBD	ctgaacggmgayctggtgt	ctgcttcatgcgcttaattaa
		gtcgcttccgtgctagca	ctgcttcatgcgcttaattaa
QMM	QX-RBD	gtcgcttccgtgctagca	tagcaatgwgtsacgaacactg
	M41-RBD	cagtgttcgtsacwcattgcta	ctgcttcatgcgcttaattaa
		gtcgcttccgtgctagca	ctgcttcatgcgcttaattaa
M41-SGS	M41-RBD	atctgatggatgtcccatcacc	ccggacttatagcaatgtgtcacg
M41-KIP	M41-RBD	tcctaagaactttctgcgggtgtc	atcttgccggtgatgggacatcc
M41-SGS-KIP	M-SGS	tcctaagaactttctgcgggtgtc	atcttgccggtgatgggacatcc
QX-Y	QX-RBD	ctgtcccatcaccggcaag	tacccggagctgtagcaatg
QX-MLQ	QX-RBD	gcagcgggaccacatcagaatttc	agcatgccggtgatgggacaaga
QX-Y-MLQ	Q-Y	gcagcgggaccacatcagaatttc	agcatgccggtgatgggacaaga

a R, nt A/G; K, nt G/T; M, nt A/C; Y, nt C/T; W, nt A/T; S, nt C/G.

b Underlined sequences indicate nucleotides changed to introduce the mutation.

### Production of recombinant proteins.

Recombinant RBD proteins were produced in human embryonic kidney (HEK293T) cells. In short, cells were transfected with pCD5 expression vectors using polyethylenimine (PEI) at a 1:12 (wt/wt) ratio. Cell culture supernatants were harvested after 6 days. The recombinant proteins were purified using Strep-Tactin Sepharose beads as previously described (19). Proteins were pretreated (where indicated) with PNGase F (New England Biolabs, USA) according to the manufacturer’s protocol before analysis by Western blotting using Strep-Tactin horseradish peroxidase (HRP) antibody (IBA, Germany).

### Circular dichroism.

Recombinant IBV RBD proteins were exchanged into buffer containing 10 mM sodium phosphate, pH 7.75, and diluted to 0.06 mg/ml. CD spectra were collected on a Jasco J-810 spectropolarimeter with a Peltier thermostatted fluorescence temperature controller module by accumulating 4 scans from 285 to 190 nm with a scanning speed of 10 nm/min, digital integrated time of 1 s, bandwidth of 1 nm, and standard sensitivity of 25°C. A thermal melt was done from 25°C to 95°C with a ramp rate of 1°C per minute. A full CD scan was collected at 95°C. After lowering the temperature to 25°C, the protein was allowed to refold for 20 min at 25°C, and a third CD scan was taken at 25°C to measure recovery. Secondary structure calculations for the CD data collected at 25°C before the thermal melt were processed by DichroWeb (23) using the CDSSTR (32), Selcon3 (33), and ContiLL (34) algorithms with protein reference set 7. Results from the 3 algorithms were averaged and plotted in Fig. 2C.

### ELISA.

Neu5Acα2-3Galβ1-3GlcNAc-PAA (Lectinity Holdings, Russia) was coated in a 96-well Nunc MaxiSorp plate (Sigma-Aldrich, Germany) at 0.5 μg/well overnight at 4°C, followed by blocking with 3% bovine serum albumin (BSA) (Sigma, Germany) in phosphate-buffered saline (PBS)-0.1% Tween 20 overnight. RBD proteins were preincubated with Strep-Tactin HRP antibody (IBA, Germany) (1:200) for 30 min on ice. Indicated protein amounts were diluted in PBS and applied onto the coated well, followed by incubation for 2 h at room temperature. TMB (3,3′,5,5′-tetramethylbenzidine; Thermo Scientific, Netherlands) substrate was used to visualize binding, after which the reaction was terminated using 1 M H_2_SO_4_. The optical density at 450 nm was measured in a FLUOstar Omega instrument (BMG Labtech), and MARS data analysis software was used for data analysis. Statistical analysis was performed using a two-way analysis of variance (ANOVA).

### Protein histochemistry.

Protein histochemistry was performed as previously described (19). Recombinant proteins precomplexed with Strep-Tactin HRP antibody (IBA, Germany) were applied onto 4-μm sections of formalin-fixed paraffin-embedded healthy chicken tissues at 100 μg/ml (for RBD, S1 and ED in equal molar amount) (24), and binding was visualized using 3-amino-9-ethylcarbazole (AEC) (Sigma-Aldrich, Germany). Where indicated, RBD proteins were preincubated with 100 μg/ml Neu5Acα2-3Galβ1-3GlcNAc (Lectinity Holdings, Russia) for 30 min on ice before application onto the tissues. Pretreatment of tissues was performed using 2 mU of neuraminidase (sialidase) from Arthrobacter ureafaciens (AUNA) (Sigma, Germany) in 10 mM potassium acetate and 2.5 mg/ml Triton X-100, pH 4.2, and incubated at 37°C overnight before protein application.
